# An Unusual Presentation of Vogt–Koyanagi–Harada

**DOI:** 10.18502/jovr.v16i1.8263

**Published:** 2021-01-20

**Authors:** Sefik Can Ipek, Sadettin Uslu, Gercek Can, Ozlem Ozbagcivan, Pinar Cakar Ozdal, Ali Osman Saatci

**Affiliations:** ^1^Department of Ophthalmology, Dokuz Eylul University, Izmir, Turkey; ^2^Department of Rheumatology, Dokuz Eylul University, Izmir, Turkey; ^3^Department of Dermatology, Dokuz Eylul University, Izmir, Turkey; ^4^Ulucanlar Eye Research and Training Hospital, University of Health Sciences, Ankara, Turkey

##  PRESENTATION 

**Figure 1:**
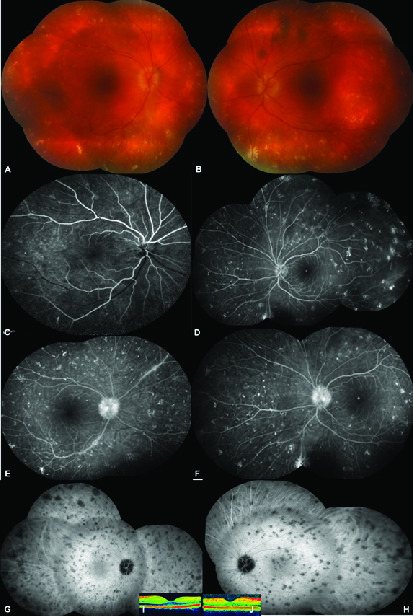
Composite of color fundus showing 360° scattered yellowish–gray spot like aggregates, (A) right eye, (B) left eye. Early venous phase of composite fluorescein angiographic picture exhibiting staining of these spot-like lesions, (C) right eye, (D) left eye. Late venous phase of composite fluorescein angiographic picture showing leakage from the optic disc, (E) (right eye), (F) left eye. Mid-phase of composite indocyanine green picture demonstrating the hypocyanescent widespread spot-like opacities, (G) right eye, (H) left eye. Normal foveal contour on optical coherence tomography, (I) right eye, (J) left eye.

**Figure 2:**
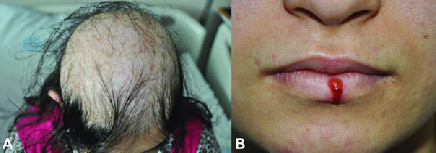
(A) Appearance of hair scalp from above depicting the diffuse alopecia. (B) Colored picture of coexistent Herpes Labialis.

**Figure 3:**
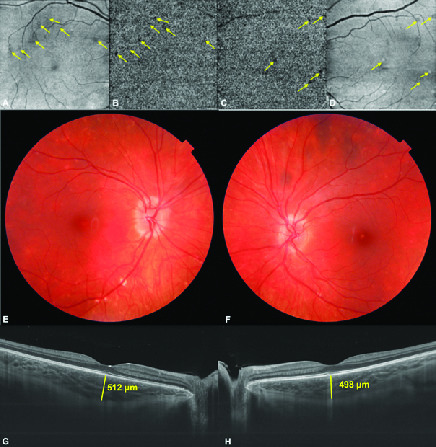
(A&B) Choriocapillaris slab of montage optical coherence tomography angiography and en-face OCT image of the right. (C&D) Left eyes revealing the patchy ischemia of choriocapillaris (arrows). Color fundus pictures taken at the last visit showing slightly blurred disc margins with less apparent old scars with no new lesion. (E) Right eye and (F) left eye. (G) Swept source optical coherence tomographic subfoveal choroidal thickness was 512 μm in the right eye and (H) 498 μm in the left eye.

In February 2018, a 21-year-old otherwise healthy female patient was diagnosed with a bilateral anterior uveitis by her local physician at a small rural hospital and was treated accordingly with topical prednisolone acetate drops. The patient also complained of a severe headache. However, no further investigation was carried out at that time. Severe alopecia ensued in March and June 2018, the patient was examined by us due to the recurrence of bilateral anterior uveitis. Her visual acuity was 20/25 OU. Bilateral (++) anterior chamber cells, non-granulomatous in nature, and a few pigmented iris clumps were observed on the anterior lens capsules. According to the SUN criteria, grade 0.5+ cells were noted in the vitreous bilaterally.^[1]^ Fundus examination revealed mildly blurred disc margins and numerous scattered yellowish-gray, round spot-like changes scattered 360° throughout the fundus OU (Figures 1A and 1B). Fluorescein angiography revealed lesions exhibiting early venous hyperfluorescence with late staining associated with mild bilateral disc leakage (Figures 1C, 1D, 1E, and 1F). An indocyanine green angiogram (Heidelberg Spectralis, Heidelberg Engineering, Heidelberg, Germany) exhibited hypocyanesence of these lesions throughout the angiography sequences (Figures 1G and 1H). Most notably, no signs of serous retinal detachment were present (Figures 1I and 1J). On the other hand, diffuse type of alopecia areata was evident (Figure 2A) and the patient was referred to the dermatology and rheumatology departments. Meticulous laboratory and imaging examinations were carried out. Laboratory results showed that the erythrocyte sedimentation rate (ESR) and cytoplasmic reactive protein (CRP) were normal. Anti-nuclear antibody (ANA) was positive (homogeneous pattern; 1/100–1/320 with dilution). Tests for HLA B27, rheumatoid factor (RF), anti-citrullinated peptide (anti-CCP), extractable nuclear antigen (ENA) panel, anti-neutrophil cytoplasmic antibody, and anti-phospholipid antibody were negative. Serum protein electrophoresis results and complement levels were normal. Furthermore, cranial and orbital MRI with contrast, chest X-ray imaging, and abdominal ultrasonography were all normal. No systemic treatment was administered as the patient had developed primary herpes labialis (Figure 2B) at the systemic evaluation phase and topical steroid treatment seemed sufficient to control the anterior chamber inflammation. In October 2018, the patient experienced a bilateral non-granulomatous anterior uveitis attack without any further posterior segment changes. Optical coherence tomography angiography and en-face OCT (Figures 3A, 3B, 3C, and 3D), as well as swept source-OCT were performed (Triton, Topcon Inc., Oakland, New Jersey, USA). This revealed that the subfoveal choroidal thickness was 512 and 498 μm in the right and left eyes, respectively (Figures 3G and 3H). At this time, oral azathioprine 50 mg daily was prescribed together with topical prednisolone acetate eye drops. This case was diagnosed as incomplete Vogt–Koyanagi–Harada (VKH) syndrome^[2]^ without overt serous retinal detachment and a significant decrease in vision. The disease progressed to the convalescent stage of VKH syndrome (with alopecia, depigmentation of fundus, and peri-papillary atrophy) as no systemic treatment was given early in the course of the disease.

##  DISCUSSION

In the present case, we found that VKH syndrome can have some unusual clinical manifestations such as no initial visual loss and no overt serous retinal detachment together with the occurrence of relatively early-onset severe alopecia. As the correct diagnosis was not made by her local physician, high-dose systemic steroids and immunosuppressants were not prescribed early at the early stages of disease presentation.

Alopecia areata is a chronic, inflammatory disorder of the hair follicles that results in non-scarring, patchy hair loss of the scalp. Histopathologically, alopecia areata associated with VKH disease is characterized with prominent pigment release, suggesting that the primary target is melanocytes and that keratinocytes might also be involved.^[3]^ However, there was a very short time lapse of one month between the occurrence of bilateral uveitis and severe alopecia in the present case. In a recent study, alopecia was present in 38 of the 261 VKH patients (13.9%) in the early disease stages, whereas in 187 of the 373 VKH patients (49.6%) it occurred in the late stages.^[4]^

The presence of serous retinal detachment can be considered as a hallmark of VKH disease. In the acute disease stages of VKH, serous retinal detachment has a positive predictive value of 100.^[5]^ Yang *et al* ^[4]^ reported that serous retinal detachment was present in 87.9% of their patients during the early stages of the disease. Remarkably, serous retinal detachment was not detected in any visits during the disease course in our case and we believe that this is very unusual in VKH disease.

Systemic high-dose corticosteroid is the first-line treatment for VKH as posterior segment involvement is almost always severe at the initiation.^[6]^ However, we examined the patient four months after the first episode of bilateral anterior uveitis. During the second anterior uveitis attack, the disease was in the convalescent stage. We decided to withhold systemic steroid and immunosuppressive therapy due to the presence of coexistent herpes labialis and the relative inactivity of the posterior segment lesions. Systemic azathioprine was prescribed four months after our initial evaluation as soon as the patient experienced the third attack of bilateral anterior uveitis.

In summary, this report presents an unusual case of VKH disease with early onset severe alopecia and the lack of overt bilateral serous retinal detachment during the early stages.

##  Financial Support and Sponsorship

Nil.

##  Conflicts of Interest

There are no conflicts of interest.
